# Integrated Assessment of Obesity Indices and Novel Inflammatory Biomarkers in Predicting the Severity of Obstructive Sleep Apnea

**DOI:** 10.3390/jcm15010273

**Published:** 2025-12-29

**Authors:** Burcu Baran, Filiz Miraç Şimşek, Hasan Durmuş, Nur Aleyna Yetkin, Bilal Rabahoğlu, Nuri Tutar, İnci Gülmez, Fatma Sema Oymak

**Affiliations:** Department of Respiratory Diseases, Erciyes University Medical School, Kayseri 38039, Turkey; dr.filizdemirbilek@gmail.com (F.M.Ş.); hasandurmus@erciyes.edu.tr (H.D.); alleynakemik@gmail.com (N.A.Y.); brabah91@hotmail.com (B.R.); drnuritutar@gmail.com (N.T.); incigul@erciyes.edu.tr (İ.G.); fsoymak@gmail.com (F.S.O.)

**Keywords:** body mass index, hypoxemia, inflammation, obstructive sleep apnea, prognostic nutritional index, tri-ponderal mass index

## Abstract

**Background/Objectives**: Obesity is a significant risk factor for obstructive sleep apnea (OSA); however, conventional anthropometric measures, such as body mass index (BMI), may not fully reflect the physiological burden associated with adiposity. The triponderal mass index (TMI) has been proposed as an alternative anthropometric indicator, while inflammation-related biomarkers have emerged as potential complementary tools for characterizing OSA severity. This study aimed to evaluate the relationships between BMI, TMI, hypoxemia, and systemic inflammation, and to assess whether combining anthropometric indices with inflammatory biomarkers improves the identification of severe OSA. **Methods**: In this retrospective cross-sectional study, 238 adults undergoing full-night polysomnography were classified into four groups: non-OSA, mild OSA, moderate OSA, and severe OSA, based on the apnea–hypopnea index (AHI). Anthropometric indices, polysomnographic parameters, and a comprehensive panel of laboratory biomarkers—including C-reactive protein (CRP), neutrophil- and platelet-derived inflammatory indices, prognostic nutritional index (PNI), CRP-to-albumin ratio (CAR), and CRP-to-lymphocyte ratio (CLR)—were analyzed. Associations were evaluated using Spearman correlation analyses, and diagnostic performance for severe OSA (AHI ≥ 30 events/h) was assessed using receiver operating characteristic (ROC) analyses, DeLong tests, and multivariable models. **Results**: Both BMI and TMI increased progressively with OSA severity (both *p* < 0.001) and showed comparable correlations with AHI and nocturnal oxygenation parameters. ROC analyses demonstrated similar discriminative performance for severe OSA (BMI AUC = 0.834; TMI AUC = 0.823; *p* = 0.229). Among inflammatory biomarkers, CRP, multi-inflammatory index (MII), CAR, and CLR showed moderate diagnostic accuracy. Among the evaluated markers, serum albumin (AUC = 0.836) and PNI demonstrated the highest diagnostic accuracy (AUC = 0.994). A combined model integrating BMI or TMI with PNI achieved near-perfect discrimination for severe OSA (BMI-based AUC = 0.9956; TMI-based AUC = 0.9969), while the addition of CRP-based inflammatory markers did not yield meaningful incremental benefit. **Conclusions**: BMI and TMI exhibit comparable performance in relation to OSA severity, hypoxemia, and systemic inflammation, with no clear superiority of TMI over BMI in adult patients. Inflammation-related biomarkers—particularly PNI—provide additional discriminatory value beyond anthropometric measures alone. Integrating simple biochemical markers with anthropometric and polysomnographic parameters may enhance risk stratification and identification of severe OSA phenotypes.

## 1. Introduction

Obstructive sleep apnea (OSA) is a highly prevalent sleep-related breathing disorder characterised by recurrent episodes of upper airway collapse during sleep, which commonly result in intermittent hypoxia and arousals. Large-scale population-based studies conducted across diverse geographic and ethnic backgrounds have reported remarkably consistent prevalence rates, with an estimated 4% of adult men and 2% of women affected [1]. The apnea–hypopnea index (AHI), representing the number of apneas and hypopneas per hour of sleep, remains the cornerstone metric for diagnosing and grading the severity of OSA [2]. Apnea is defined as a complete cessation of airflow lasting at least 10 s, whereas hypopnea entails a partial reduction in airflow accompanied by ≥3% oxygen desaturation or an arousal. These recurrent respiratory disturbances lead to cyclical hypoxemia and contribute to downstream pathophysiological effects. Major risk factors for OSA in adults include advancing age, obesity (defined by a body mass index [BMI] of ≥30 kg/m^2^), and an increased neck circumference (>43 cm in men and >38 cm in women) [3].

Obesity, a chronic low-grade inflammatory state characterized by excessive adipose tissue accumulation, is a major contributor to OSA pathogenesis through both mechanical and metabolic mechanisms. Nevertheless, OSA may also occur in individuals without overt obesity, and previous studies have demonstrated associations between sleep apnea and other cardiometabolic abnormalities, including dyslipidemia, hypertension, increased body surface area, and impaired glucose tolerance [4].

BMI is the most widely used measure to assess obesity and has been shown to correlate with the severity of OSA [5]. The triponderal mass index (TMI), calculated as weight divided by height cubed (kg/m^3^), has emerged as a more height-independent indicator of adiposity. Although initially developed for pediatric populations, TMI has shown promising performance in adults for predicting metabolic and cardiopulmonary risk [6,7,8,9]. In the context of OSA, Yetkin et al. reported that TMI was more strongly correlated with disease severity than BMI, suggesting its potential role as an alternative anthropometric marker in OSA risk stratification [9]. Beyond mechanical factors, systemic inflammation has been proposed as a key contributor to the pathophysiology of OSA. Inflammatory responses, which play a central role in numerous chronic diseases [10,11,12,13], have also been demonstrated in OSA. Histopathological studies of uvular tissue from OSA patients have revealed subepithelial oedema and inflammatory cell infiltration [14]. A meta-analysis by Nadeem et al. [15] confirmed significantly elevated circulating levels of C-reactive protein (CRP), tumour necrosis factor-α, and interleukin-6 in affected individuals. In addition, composite inflammatory indices, such as the systemic immune-inflammation index (SII), have been associated with OSA severity and may offer prognostic value. Collectively, these findings highlight the complex relationship between obesity, systemic inflammation, and cardiovascular risk associated with OSA. More recently, growing attention has focused on novel inflammation-related biomarkers derived from routine laboratory parameters, including neutrophil-to-lymphocyte ratio (NLR), platelet-to-lymphocyte ratio (PLR), monocyte-to-lymphocyte ratio (MLR), SII, multi-inflammatory index (MII), pan-immune–inflammation value (PIV), prognostic nutritional index (PNI), CRP-to-lymphocyte ratio (CLR), and CRP-to-albumin ratio (CAR), which have demonstrated prognostic relevance across a wide range of inflammation-related conditions [16,17,18,19,20].

This study aimed to comprehensively evaluate the relationships between obesity-related anthropometric indices and OSA severity, with particular emphasis on comparing BMI and TMI in relation to hypoxemia and systemic inflammation. In addition, we aimed to evaluate the diagnostic performance of routinely available inflammation-related biomarkers and to determine whether the combined assessment of anthropometric indices and inflammatory markers enhances the prediction and discrimination of severe OSA.

## 2. Materials and Methods

### 2.1. Data Source and Inclusion Criteria

This retrospective, cross-sectional study was conducted at the Sleep Disorders Unit of Erciyes University Faculty of Medicine Hospital between January and December 2024. Adult patients (aged 18 years or older) who underwent full-night polysomnography (PSG) for suspected OSA during the study period were screened for eligibility. Based on the American Academy of Sleep Medicine (AASM) criteria, participants were classified according to AHI into four groups: non-OSA (AHI < 5 events/h), mild OSA (5 ≤ AHI < 15 events/h), moderate OSA (15 ≤ AHI < 30 events/h), and severe OSA (AHI ≥ 30 events/h) [2].

Eligible participants were required to have complete anthropometric measurements, as well as full blood count and biochemical analyses obtained during the same hospitalisation period, with blood samples collected in the morning immediately after awakening, following overnight PSG. Patients were excluded if they had: (1) active or chronic systemic inflammatory diseases (including rheumatoid arthritis, systemic lupus erythematosus, inflammatory bowel disease, malignancy, chronic liver disease, advanced kidney failure, or New York Heart Association class III–IV heart failure); (2) clinical or laboratory evidence of acute infection at the time of PSG or within the preceding four weeks (such as fever, leukocytosis, elevated C-reactive protein levels, or a documented infectious diagnosis); (3) incomplete polysomnographic, anthropometric, or laboratory data; or (4) a history of prior OSA treatment, including continuous positive airway pressure (CPAP) therapy or upper airway surgery.

**Figure 1:**
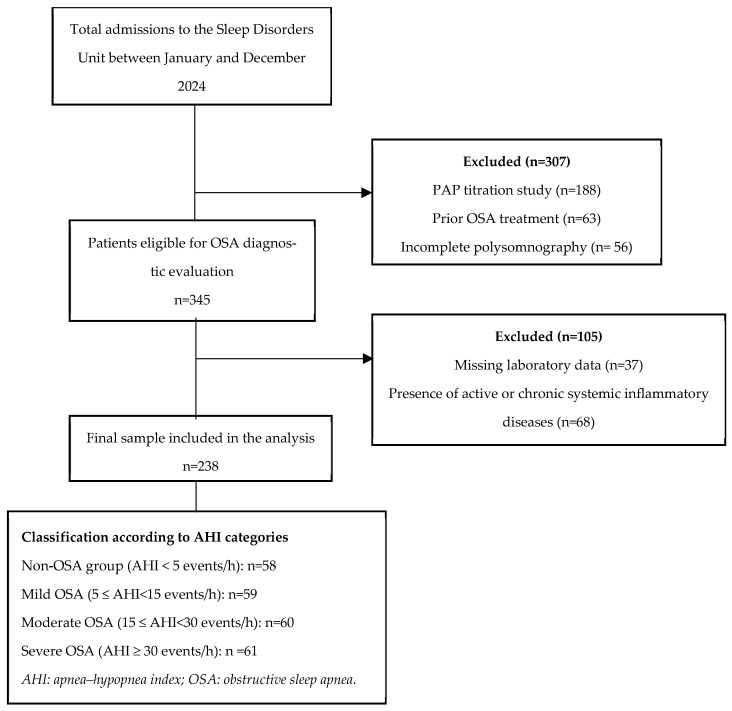
Study flow diagram.

The participant selection process, inclusion and exclusion criteria, and final study population are summarised in the study flow diagram (Figure 1).

### 2.2. Anthropometric Measurements

Height and body weight were measured in all participants during the morning hours following an overnight fast, with subjects wearing light clothing and no shoes. BMI was calculated as weight divided by height squared (kg/m^2^), while TMI was calculated as weight divided by height cubed (kg/m^3^) [6]. For subgroup analyses, obesity was defined as having a BMI ≥ 30 kg/m^2^ and TMI ≥ 17 kg/m^3^. These thresholds were selected based on prior literature, as well as receiver operating characteristic (ROC) curve analyses performed within the present cohort, to determine the optimal cut-off values associated with severe OSA.

### 2.3. Polysomnographic Assessment

All participants underwent a full-night attended PSG. Recordings were performed using a 64-channel Compumedics E-Series system (Victoria, Australia). The evaluation included four-channel electroencephalography, electrooculography, submental and tibial electromyography, electrocardiography, body position monitoring, nasal airflow measurement, thoracoabdominal respiratory effort monitoring, and fingertip pulse oximetry for SpO_2_ measurement. Key parameters recorded included total sleep time, sleep efficiency, oxygen desaturation index (ODI), minimum oxygen saturation (minSpO_2_), mean oxygen saturation (meanSpO_2_), and AHI. The diagnosis of OSA was established according to the criteria of the International Classification of Sleep Disorders, Third Edition [21]

### 2.4. Laboratory Analysis and Inflammatory Biomarkers

Following PSG, venous blood samples obtained during the same hospitalization period were analyzed to assess complete blood count parameters (including leukocyte, neutrophil, lymphocyte, monocyte, and platelet counts, haemoglobin, and mean platelet volume [MPV]), CRP, and albumin levels. Based on these variables, the following inflammatory biomarkers were calculated:

- NLR: Neutrophil-to-lymphocyte ratio
- PLR: Platelet-to-lymphocyte ratio
- MLR: Monocyte-to-lymphocyte ratio
- SII: Platelet count × NLR
- MII: (Neutrophil × monocyte)/lymphocyte
- PIV: (Neutrophil × monocyte × platelet)/lymphocyte
- PNI: Albumin (g/L) + 5 × lymphocyte count (109/L)
- CAR: CRP/albumin
- CLR: CRP/lymphocyte count.

All laboratory analyses were performed using Pentra Nexus (Horiba, Kyoto, Japan) and Sysmex XN (Sysmex Corporation, Kobe, Japan) analyzers.

### 2.5. Statistical Analysis

Statistical analyses were performed using SPSS Statistics version 22.0 (IBM Corp., Armonk, NY, USA) and R software (version 4.3.0; R Foundation for Statistical Computing, Vienna, Austria). The normality of continuous variables was assessed using the Shapiro–Wilk test. Normally distributed data are presented as mean ± standard deviation, whereas non-normally distributed variables are reported as median (minimum–maximum). Categorical variables are expressed as frequencies and percentages.

Comparisons among OSA severity groups were conducted using one-way analysis of variance (ANOVA) for normally distributed variables or the Kruskal–Wallis test for non-normally distributed variables, with Bonferroni-adjusted post hoc pairwise comparisons where appropriate. Categorical variables were compared using the chi-square test or Fisher’s exact test.

Correlations between anthropometric indices, polysomnographic parameters, and inflammation-based biomarkers were evaluated using Spearman’s rank correlation coefficient (ρ). To control for multiple testing across correlation analyses, *p*-values were adjusted using the Benjamini–Hochberg false discovery rate (FDR) procedure. Ninety-five per cent confidence intervals (95% CI) were calculated for statistically significant correlations.

ROC curve analyses were performed to assess the ability of anthropometric indices and inflammation-related biomarkers to distinguish between severe OSA, defined as an AHI of 30 events/h or more, and non-severe OSA. For each marker, the area under the ROC curve (AUC) with 95% CI, sensitivity, specificity, positive predictive value (PPV), and negative predictive value (NPV) were calculated. Optimal cut-off values were determined using the Youden index. Comparisons between correlated ROC curves derived from the same study population were performed using DeLong’s test. Combined models were constructed using selected anthropometric and inflammation-related markers based on biological plausibility and clinical interpretability, with care taken to avoid collinearity and redundancy among composite indices.

In addition to single-marker analyses, combined predictive models were constructed using multivariable logistic regression based on predefined clinical and biological considerations. Two model structures were evaluated: Model A combined an anthropometric index with PNI, whereas Model B additionally incorporated markers of systemic inflammation, including CRP and CAR. To assess the relative contribution of anthropometric indices, both BMI-based and TMI-based versions of each model were developed. Predicted probabilities from these models were used to generate ROC curves, and model performances were compared using DeLong’s test.

Given the retrospective design, post hoc power analysis was conducted using G*Power software (version 3.1). Effect sizes (Cohen’s f) were derived from one-way ANOVA models for key biomarkers (PNI, CRP, CAR, CLR, and TMI). The achieved statistical power exceeded 0.99 for all evaluated parameters at a two-sided α level of 0.05. A two-tailed *p*-value < 0.05 was considered statistically significant.

## 3. Results

### 3.1. Descriptive Statistics

A total of 238 participants were included in the study and were classified into four groups based on AHI: non-OSA (*n* = 58), mild OSA (*n* = 59), moderate OSA (*n* = 60), and severe OSA (*n* = 61). The mean age of the cohort was 49.3 ± 10.2 years, and 50% (*n* = 119) were male. Age differed significantly across groups, with patients in the mild OSA group being younger than those in the moderate and severe groups (*p* = 0.004). In contrast, sex distribution showed no significant variation (*p* = 0.597). Both anthropometric indices demonstrated a transparent severity-dependent gradient, with mean BMI increasing from 25.8 ± 2.7 kg/m^2^ in the non-OSA group to 38.5 ± 8.3 kg/m^2^ in the severe OSA group, paralleled by a corresponding rise in mean TMI from 15.3 ± 1.9 kg/m^3^ to 24.0 ± 6.0 kg/m^3^ (Table 1).

Among routine hematologic parameters, leukocyte, lymphocyte, monocyte, haemoglobin, and platelet counts did not differ significantly across groups. In contrast, neutrophil counts were significantly higher in patients with severe OSA than in those with moderate OSA (*p* = 0.032). MPV and serum albumin levels were also significantly lower in the severe OSA group (both *p* < 0.001). Among inflammation-related biomarkers, CRP, MII, CAR, and CLR were markedly elevated in the severe OSA group compared with the non-OSA, mild and moderate OSA groups (*p* < 0.001 for all). NLR was also significantly higher in the severe OSA group compared to the moderate OSA group (*p* = 0.040). In contrast, PNI was significantly reduced in severe OSA (*p* < 0.001). No significant intergroup differences were detected in PLR, MLR, SII, or PIV (Table 1).

Polysomnographic parameters showed clear and significant differences across OSA severity categories. Median AHI values increased progressively from 1.9 events/h (0–4.2) in the non-OSA group to 8.7 events/h (5.2–14.8) in mild OSA, 18.9 events/h (15.2–28.3) in moderate OSA, and 60.0 events/h (31.0–115.9) in severe OSA (*p* < 0.001). Median ODI values showed a similar stepwise increase, rising from 5.6 events/h (1.3–10.1) in non-OSA to 8.7 events/h (1.3–23.3) in mild OSA, 23.5 events/h (5.9–50.6) in moderate OSA, and 85.7 events/h (22.7–142.2) in severe OSA (*p* < 0.001). Both minSpO_2_ and meanSpO_2_ levels declined significantly with increasing OSA severity, whereas total sleep time and sleep efficiency remained comparable across groups (Table 1).

### 3.2. Correlation Analysis

BMI, TMI, and AHI were significantly intercorrelated (BMI–TMI: ρ = 0.960; BMI–AHI: ρ = 0.515; TMI–AHI: ρ = 0.470), with all associations remaining statistically significant after FDR correction. All three indices showed similar association patterns, exhibiting significant positive correlations with age and ODI, while both minSpO_2_ and meanSpO_2_ were negatively correlated with these measures.

Inflammation-related biomarkers, including CRP, MII, CAR, and CLR, showed significant positive correlations with BMI, TMI, and AHI, whereas albumin and PNI exhibited inverse correlations with all three parameters. These associations remained statistically significant after FDR adjustment (Table 2). In contrast, no significant correlations were observed for other hematologic or inflammatory indices following correction for multiple testing.

### 3.3. ROC Analysis

**Figure 2:**
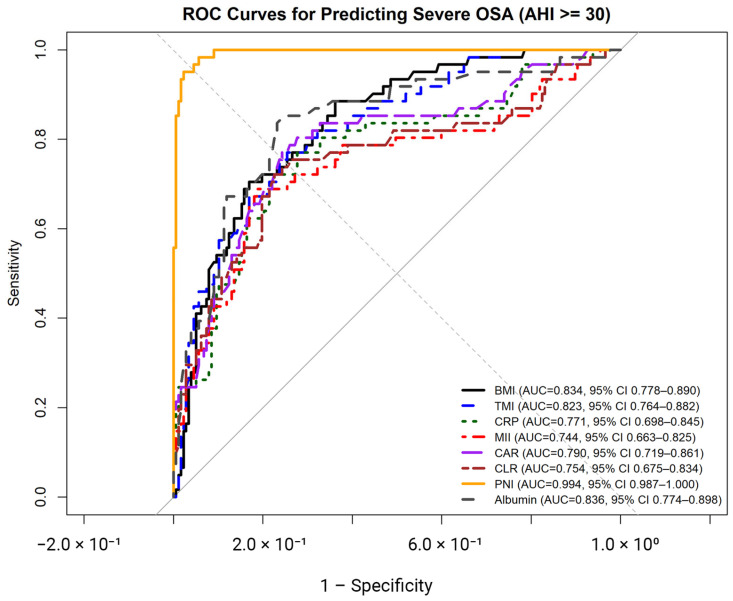
Receiver operating characteristic (ROC) curves illustrating the diagnostic performance of body mass index (BMI), triponderal mass index (TMI), C-reactive protein (CRP), multi-inflammatory index (MII), C-reactive protein–to–albumin ratio (CAR), C-reactive protein–to–lymphocyte ratio (CLR), prognostic nutritional index (PNI), and serum albumin for identifying severe obstructive sleep apnea (OSA; AHI ≥ 30 events/h).

ROC analyses were performed to evaluate the diagnostic performance of anthropometric indices and inflammation-related biomarkers for identifying severe OSA (Figure 2, Table 3). Among anthropometric indices, BMI demonstrated an AUC of 0.834 (95% CI: 0.778–0.890), with an optimal cut-off value of ≥33.3 kg/m^2^, yielding a sensitivity of 70.5% and a specificity of 83.1%. TMI showed an AUC of 0.823 (95% CI: 0.764–0.882) with an optimal cut-off of ≥19.7 kg/m^3^, corresponding to a sensitivity of 75.4% and a specificity of 75.3%. For both indices, chi-square analyses confirmed significant associations with severe OSA (both *p* < 0.001).

Among inflammation-related biomarkers, CRP, MII, CAR, and CLR demonstrated moderate diagnostic accuracy, with AUC values ranging from 0.744 to 0.791 (all *p* < 0.001). Optimal cut-off values for these markers yielded sensitivities ranging from 68.9% to 77.0% and specificities ranging from 75.7% to 81.4%, with all corresponding chi-square tests indicating significant associations with severe OSA (all *p* < 0.001).

Nutritional markers showed higher discriminative performance. Serum albumin yielded an AUC of 0.836 (95% CI: 0.774–0.898), with an optimal cut-off of ≤4.49 g/dL, resulting in a sensitivity of 83.6% and a specificity of 76.8%. PNI demonstrated the highest diagnostic accuracy, with an AUC of 0.994 (95% CI: 0.987–1.000) and an optimal cut-off of ≤48.1, corresponding to a sensitivity of 95.1% and a specificity of 97.7%. Chi-square analyses confirmed significant associations between reduced albumin and PNI levels and severe OSA (both *p* < 0.001).

Pairwise comparisons of areas under the ROC curves were performed using DeLong’s test to evaluate differences in diagnostic performance between BMI and selected anthropometric and inflammation-related biomarkers for identifying severe OSA (Table 4). No statistically significant difference was observed between BMI and TMI (ΔAUC = −0.011, Z = 1.20, *p* = 0.229). Similarly, comparisons between BMI and CRP (*p* = 0.138), BMI and CAR (*p* = 0.289), and BMI and CLR (*p* = 0.078) did not reveal statistically significant differences in AUC values. In contrast, BMI and PNI differed significantly, with PNI demonstrating a higher AUC compared with BMI (ΔAUC = −0.160, Z = −5.55, *p* < 0.001). No other pairwise comparisons reached statistical significance.

**Figure 3:**
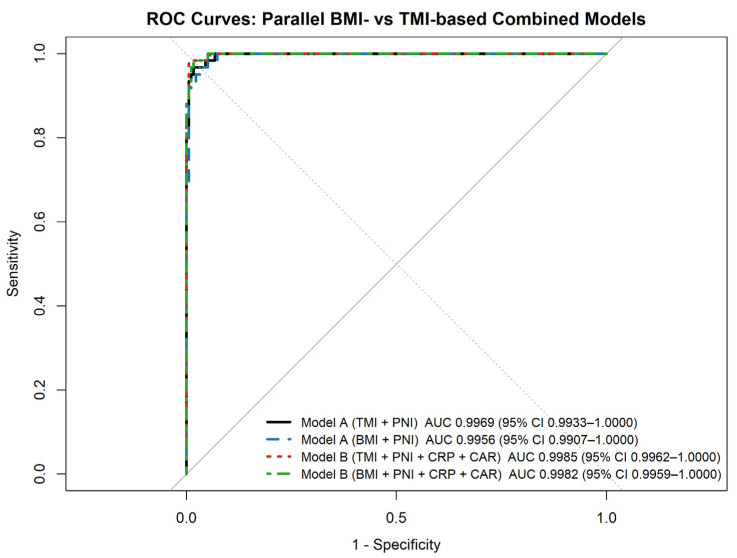
ROC curves of combined predictive models for severe obstructive sleep apnea (Receiver operating characteristic (ROC) curves illustrating the diagnostic performance of parallel BMI-based and TMI-based combined predictive models for identifying severe obstructive sleep apnea (AHI ≥ 30 events/h). Model A combines an anthropometric index (BMI or TMI) with the prognostic nutritional index (PNI). In contrast, Model B additionally incorporates inflammatory markers (C-reactive protein and C-reactive protein–to–albumin ratio). Areas under the curve (AUCs) with 95% confidence intervals are shown for each model.).

In addition to single-marker analyses, parallel BMI-based and TMI-based combined predictive models were evaluated for discriminating severe OSA (Figure 3). In Model A, which combined an anthropometric index with PNI, both the TMI-based and BMI-based formulations demonstrated excellent diagnostic performance (AUC = 0.9969 and 0.9956, respectively). DeLong’s test showed no statistically significant difference between the BMI-based and TMI-based versions of Model A.

Similarly, Model B, which additionally incorporated inflammatory markers (CRP and CAR), yielded very high discriminatory ability in both formulations (TMI-based AUC = 0.9985; BMI-based AUC = 0.9982), with no significant difference between anthropometric indices. For both model structures, TMI-based and BMI-based formulations demonstrated comparable discriminative performance when combined with nutritional and inflammatory biomarkers. The addition of inflammatory markers in Model B did not result in a statistically significant improvement over Model A.

### 3.4. BMI- and TMI-Based Comparisons

Patients were stratified into two subgroups based on BMI (<30 vs. ≥30 kg/m^2^) and TMI (<17 vs. ≥17 kg/m^3^). In both classifications, individuals with higher BMI and TMI values were significantly older (*p* < 0.001) and more frequently female (*p* < 0.001). In the BMI-based analysis, patients with BMI ≥30 kg/m^2^ demonstrated significantly higher AHI and ODI values (both *p* < 0.001), accompanied by markedly lower meanSpO_2_ and minSpO_2_ levels (*p* < 0.001 for each parameter). A similar pattern was observed in the TMI-based comparison: subjects with TMI ≥17 kg/m^3^ exhibited higher AHI and ODI values and reduced oxygenation indices (all *p* < 0.001). With respect to hematologic and inflammatory markers, CRP, MII, CAR, and CLR were substantially elevated in patients with increased BMI and TMI (*p* < 0.001 for all). In contrast, haemoglobin, serum albumin, and PNI values were significantly lower in groups with more severe obesity (*p* < 0.001 for all) (Table 5).

## 4. Discussion

In this study, both BMI and TMI values increased progressively with OSA severity, reflecting a strong association between obesity and disease burden. Patients with higher BMI and TMI values were significantly older, and sex distribution differed across severity categories. Severe OSA was characterized by markedly elevated levels of inflammation-related biomarkers, including CRP, MII, CAR, and CLR, accompanied by significant reductions in MPV, albumin and PNI, indicating concurrent inflammatory activation and alterations in nutritional status with increasing disease severity.

Correlation analyses demonstrated that BMI, TMI, and AHI were strongly interrelated and exhibited broadly similar association patterns with inflammatory and oxygenation-related parameters. All three indices showed positive correlations with CRP, MII, CAR, and CLR, and inverse correlations with albumin and PNI. ROC analyses further demonstrated that BMI and TMI showed comparable diagnostic performance for identifying severe OSA, with no statistically significant difference between their AUC values on DeLong testing. In contrast, nutritional markers—most notably serum albumin and PNI—demonstrated higher discriminative performance in single-marker ROC analyses, with PNI significantly outperforming BMI. Consistent with these findings, a combined model integrating BMI or TMI with PNI (Model A) achieved very high discriminative performance, while the addition of inflammatory biomarkers did not meaningfully improve model accuracy.

OSA is a major global public health problem that closely parallels the worldwide rise in obesity, and population-based studies have consistently identified obesity as one of its strongest risk factors, with a precise dose–response relationship between adiposity and disease severity [1,22]. In a meta-analysis including 12,860 individuals, OSA prevalence (AHI ≥ 5 events/h) was 56.2%, while obesity (BMI ≥ 30 kg/m^2^) was present in 25.7% of the general population; notably, the proportion of obesity among individuals with OSA increased progressively with AHI severity, reaching 47.2% in severe disease (AHI ≥ 30 events/h) [22]. Consistent with the growing recognition of obesity as a multidimensional and dynamically evolving health risk, reflected by recent machine learning-based obesity risk prediction models that emphasize individualized risk stratification, accurate anthropometric characterization remains central to OSA research and clinical practice [23]. In line with these observations, the present study demonstrated an apparent severity-dependent increase in both BMI- and TMI-defined obesity, with comparable prevalence estimates across the OSA cohort.

TMI was initially proposed as a more stable anthropometric measure of adiposity, particularly in younger populations, where it correlates more closely with body fat percentage and metabolic risk than BMI [6,7,8,24,25]. However, its role in adult OSA populations has remained insufficiently unexplored. In the present cohort, TMI did not demonstrate superiority over BMI; instead, both indices increased progressively with OSA severity and showed comparable associations with hypoxemia-related parameters, supported by similar ROC performance. These findings are consistent with recent adult OSA studies, including that of Yetkin et al., which reported significant associations between TMI and OSA severity without clear superiority over BMI [9]. Nevertheless, the consistent relationship between TMI and disease severity supports its use as an alternative surrogate of adiposity in clinical contexts where BMI may inadequately reflect body composition.

In the present study, BMI, TMI, and AHI were strongly interrelated and exhibited comparable association patterns with nocturnal oxygenation parameters. All three measures showed significant positive correlations with ODI and significant negative correlations with both meanSpO_2_ and minSpO_2_, indicating that a parallel deterioration in nocturnal oxygenation accompanies increasing adiposity and disease severity. These findings are consistent with extensive epidemiological studies, which demonstrate that higher obesity indices are associated with deeper and more frequent desaturation events during sleep [3,22]. Similarly, Wali et al. reported significant negative correlations between BMI and both minSpO and meanSpO_2_ (ρ = −0.44 and −0.39; *p* <0.001) [26], while Uzair et al. observed an association between BMI and nadir SpO_2_ independent of AHI [27]. Collectively, these observations support the concept that obesity-related upper airway collapsibility contributes to more profound hypoxemia in OSA, thereby reinforcing the close interplay between adiposity, intermittent hypoxia, and downstream pathophysiological processes.

Systemic inflammation represents a key mechanism linking intermittent hypoxia to OSA-related comorbidities. Recurrent hypoxia–reoxygenation cycles induce oxidative stress and activation of inflammatory pathways [14,15]. In this study, CRP, CAR, CLR, and MII were significantly elevated in patients with severe OSA and showed consistent correlations with BMI, TMI, and AHI, exhibiting moderate but overlapping diagnostic performance. CRP remains the most extensively studied inflammatory marker in OSA, and prior meta-analyses and extensive cohort studies have consistently demonstrated higher CRP levels in patients with more severe disease, particularly in the presence of obesity [15,20,28]. In a meta-analysis by Nadeem et al., serum CRP levels were significantly elevated in patients with OSA compared with controls (standardized mean difference = 1.77) [15]. Similarly, cohort data indicate that severe OSA independently increases the likelihood of CRP > 3 mg/L, with this association being further amplified by coexisting obesity [20]. This threshold closely aligns with our ROC-derived cut-off for CRP, supporting its clinical relevance in identifying severe OSA. A recent systematic review further confirmed higher CRP and high-sensitivity CRP levels in OSA, with more pronounced elevations among obese individuals [28].

CRP-based composite indices, such as CAR and CLR, have been proposed as integrative markers reflecting inflammatory activity in conjunction with nutritional or immune status [12,18]. Hizli et al. reported significantly higher CAR levels in moderate-to-severe OSA compared with controls, even when traditional cell count–derived indices were not consistently altered, a finding that parallels our results [18]. Population-based data further support the sensitivity of CAR to adiposity-related inflammation [29]. Evidence for CLR in OSA remains limited; however, studies of the lymphocyte-to-CRP ratio report modest discriminative performance (AUC = 0.683; 95% CI: 0.600–0.759), comparable to that observed in our cohort [4]. MII, a composite marker of innate immune activation [10,11,13], was elevated in severe OSA and correlated with BMI, TMI, and AHI; however, it demonstrated only moderate discriminative ability. Given its overlap with other inflammatory markers and lack of incremental diagnostic value, MII was not retained in multivariable modelling.

Markers reflecting nutritional status, particularly serum albumin and PNI, showed a distinct pattern compared with anthropometric and inflammation-based indices. Albumin is closely linked to nocturnal hypoxemia rather than apnea frequency per se, supporting the concept that chronic intermittent hypoxia directly affects albumin metabolism and redox balance [30,31]. PNI, which integrates serum albumin with lymphocyte count, may therefore capture both hypoxia-related nutritional impairment and immune dysregulation. Although PNI has not been previously evaluated in OSA, extensive evidence from oncologic, infectious, and inflammatory conditions demonstrates its sensitivity to systemic inflammation and adverse clinical phenotypes [16,17,19,32]. In the context of OSA, hypoxia-driven inflammatory activation and impaired albumin synthesis provide a biologically plausible explanation for the strong discriminatory performance observed with PNI (AUC = 0.994; optimal cut-off ≤ 48.1), highlighting nutritional–immune imbalance as a previously underrecognized dimension of severe disease [31,33].

Given the exceptionally high discriminatory performance of PNI in single-marker analyses, multivariable modelling was performed to determine whether integrating nutritional status with anthropometric and inflammatory parameters would provide incremental value for identifying severe OSA while preserving model parsimony. In this framework, Model A, combining BMI or TMI with PNI, achieved near-perfect discrimination for severe disease (BMI-based AUC = 0.9956; TMI-based AUC = 0.9969), whereas the addition of CRP-based inflammatory markers in Model B did not yield a clinically meaningful improvement. BMI- and TMI-based models performed comparably across both structures. Previous combined predictive models for moderate-to-severe or severe OSA have primarily integrated anthropometric and clinical variables. For instance, a nomogram-based regression model incorporating BMI, hypertension, key nocturnal symptoms, and Epworth Sleepiness Scale score demonstrated high discriminative performance for moderate-to-severe OSA (AUC = 0.976; 95% CI: 0.962–0.990; sensitivity 95.9%, specificity 89.7%) [34]. However, systemic inflammatory and nutritional dimensions have rarely been incorporated, and nutritional–immune indices such as PNI remain largely unexplored in this context [18,20].

Among additional hematologic parameters, MPV was modestly reduced in patients with severe OSA and showed a weak inverse correlation with AHI, consistent with previous reports describing heterogeneous MPV responses in this setting [33,35]. In contrast, other cell count–derived inflammatory indices, including NLR, PLR, MLR, SII, and PIV, did not differ significantly across OSA severity categories and were not meaningfully associated with AHI or adiposity indices, suggesting limited utility for reflecting OSA-specific inflammatory burden within this cohort.

This study has several limitations that should be taken into account when interpreting the findings. First, the retrospective and cross-sectional design precludes causal inference between anthropometric indices, inflammatory biomarkers, and OSA severity. Although rigorous exclusion criteria were applied to minimize confounding—particularly by acute infection, chronic inflammatory disease, malignancy, and advanced organ failure—residual confounding related to unmeasured metabolic, dietary, or lifestyle factors cannot be entirely ruled out. Second, a separate healthy control group was not included, as polysomnography is not routinely performed in asymptomatic individuals for research purposes alone. Instead, the non-OSA group comprised patients referred for polysomnography due to sleep-related symptoms but classified as non-OSA based on an AHI < 5 events/h, reflecting real-world clinical practice.

Nevertheless, the presence of symptoms in this group may have been associated with mild nocturnal desaturation or subclinical inflammatory changes, which could potentially attenuate between-group differences. Third, the single-centre nature of the study may limit generalizability to populations with different demographic characteristics, comorbidity profiles, or referral patterns. In addition, although both BMI and TMI were evaluated, interpretation of TMI in adults remains constrained by the absence of universally accepted reference ranges and validated age- and sex-specific cut-off values, which may limit its immediate clinical applicability despite its comparable performance to BMI. Fourth, although a broad panel of inflammatory biomarkers was evaluated, serum albumin and the prognostic nutritional index may have been influenced by nutritional status or unmeasured metabolic or inflammatory factors. Finally, despite adequate post hoc statistical power, the retrospective design and absence of external validation warrant cautious interpretation of the high discriminative performance observed for PNI and the combined models, pending external validation.

## 5. Conclusions

In this study, both BMI and TMI increased progressively with OSA severity and showed comparable associations with hypoxemia and systemic inflammation, with no clear superiority of TMI over BMI in adult patients. Nevertheless, the similar diagnostic performance of TMI supports its use as an alternative anthropometric measure in selected clinical contexts. Inflammatory and nutritional biomarkers, particularly CRP-based indices, serum albumin, and PNI, demonstrated strong associations with disease severity, with PNI showing the highest discriminative performance. Given their wide availability and low cost, these markers may provide complementary information beyond AHI for identifying high-risk OSA phenotypes. Overall, integrating simple biochemical markers with anthropometric and polysomnographic parameters may improve clinical risk stratification in OSA. Future multicenter studies with external validation are needed to confirm these findings.

## Figures and Tables

**Table 1:** Demographic, laboratory, inflammatory, and polysomnographic biomarkers across non-OSA and OSA severity groups.

	Non-OSA *n* = 58	Mild OSA *n* = 59	Moderate OSA *n* = 60	Severe OSA *n* = 61	*p*-Value
Male sex, *n* (%)	30 (52)	32 (54)	31 (52)	26 (43)	0.597
Age (years)	48.2 ± 8.8 ^ab^	45.4 ± 10.7 ^a^	51 ± 9.9 ^b^	51.4 ± 10.4 ^b^	0.004
BMI (kg/m^2^) TMI (kg/m^3^)	25.8 ± 2.7 ^a^ 15.3 ± 1.9 ^a^	29.1 ± 4.2 ^b^ 17.5 ± 2.9 ^b^	34.2 ± 11.3 ^c^ 20.4 ± 5.7 ^c^	38.5 ± 8.3 ^d^ 24.0 ± 6.0 ^d^	<0.001 <0.001
Laboratory values, Leukocyte count (×10^9^/L) Neutrophil count (×10^9^/L) Lymphocyte count (×10^9^/L) Monocyte count (×10^9^/L) Haemoglobin (g/dL) Platelet count (×10^9^/L) MPV (fL) Albumin (g/dL)	7.3 (4.0–10.4) 4.3 (1.8–7.7) ^ab^ 2.3 (0.5–4.3) 0.5 (0.3–1.1) 14.6 ± 1.7 276 ± 61 10.2 (7.2–12.3) ^a^ 4.60 ± 0.38 ^a^	7.4 (4.4–10.5) 4.2 (2.1–7.9) ^ab^ 2.3 (0.9–4.6) 0.6 (0.3–1.3) 15.2 ± 1.6 256 ± 66 10.2 (8.9–12.5) ^a^ 4.71 ± 0.27 ^a^	7.2 (3.9–13.9) 4.1 (1.9–9) ^a^ 2.4 (1.2–4.7) 0.5 (0.2–1) 15 ± 1.8 271 ± 58 10.3 (7.6–12.2) ^a^ 4.68 ± 0.24 ^a^	7.6 (4.4–17.2) 4.8 (2.3–14.5) ^b^ 2.2 (0.6–6.2) 0.5 (0.3–1.4) 14.6 ± 1.9 255 ± 59 9.8 (6.8–11.5) ^b^ 4.20 ± 0.39 ^b^	0.052 0.042 0.423 0.334 0.278 0.148 <0.001 <0.001
Inflammatory biomarkers, CRP (mg/L) NLR PLR MLR SII MII PIV PNI CAR CLR	2.1 (0.2–10.7) ^a^ 1.8 (1.0–9.9) ^ab^ 116 (54–335) 0.2 (0.1–0.9) 490 (191–1823) 882 (103–17,880) ^a^ 272 (83–1162) 57.2 ± 5.4 ^a^ 0.45 (0.04–3.13) ^a^ 0.92 (0.09–9.85) ^a^	2.3 (0.2–6.7) ^a^ 2.0 (0.8–8.2) ^ab^ 112 (49–258) 0.2 (0.1–0.7) 478 (210–1804) 1061 (76–8459) ^a^ 258 (63–1263) 58.4 ± 4.7 ^a^ 0.46 (0.04–1.48) ^a^ 0.89 (0.07–4.90) ^a^	2.2 (0.2–13) ^a^ 1.7 (0.8–5.2) ^a^ 111 (55–283) 0.2 (0.1–0.4) 444 (182–1279) 969 (96–11,961) ^a^ 223 (74–1035) 58.9 ± 4 ^a^ 0.46 (0.06–2.8) ^a^ 0.87 (0.09–6.28) ^a^	5.6 (0.5–61.8) ^b^ 2.0 (0.7–17.7) ^b^ 107 (42–502) 0.2 (0.1–1.1) 526 (166–4873) 2999 (146–44,930) ^b^ 290 (79–2652) 42 ± 3.9 ^b^ 1.3 (0.12–14.72) ^b^ 2.9 (0.2–26.8) ^b^	<0.001 0.040 0.344 0.439 0.301 <0.001 0.245 <0.001 <0.001 <0.001
Sleep Parameters					
Total sleep time (min)	284 ± 58	281 ± 64	280 ± 58	286 ± 79	0.532
Sleep efficiency (%)	79 ± 16	74 ± 18	76 ± 16	78 ± 15	0.322
AHI (events/h)	1.9 (0–4.2) ^a^	8.7 (5.2–14) ^b^	18.9 (15.1–28.3) ^c^	60 (31–115.9) ^d^	<0.001
ODI (events/h)	5.6 (1.3–10.1) ^a^	8.7 (1.3–23.3) ^b^	23.3 (5.9–50.6) ^c^	85.7 (22.7–142.2) ^d^	<0.001
Minimum SpO_2_ (%)	92 (88–94) ^a^	86 (72–92) ^b^	82 (63–89) ^c^	65 (50–87) ^d^	<0.001
Mean SpO_2_ (%)	93 (90–96) ^a^	92 (89–96) ^b^	90 (86–94) ^c^	83 (58–96) ^d^	<0.001

Data are presented as mean ± standard deviation for normally distributed variables and median (minimum–maximum) for non-normally distributed variables. Comparisons among non-OSA, mild, moderate, and severe OSA groups were performed using the Kruskal–Wallis test, followed by Dunn’s post hoc test with Bonferroni correction for pairwise comparisons. Superscript letters (^a–d^) indicate statistically significant differences between groups. A *p*-value < 0.05 was considered statistically significant. AHI—apnea–hypopnea index; BMI—body mass index; CAR—C-reactive protein to albumin ratio; CLR—C-reactive protein to lymphocyte ratio; CRP—C-reactive protein; MII—multi-inflammatory index; MLR—monocyte-to-lymphocyte ratio; MPV—mean platelet volume; NLR—neutrophil-to-lymphocyte ratio; ODI—oxygen desaturation index; OSA—obstructive sleep apnea; PIV—pan-immune—inflammation value; PLR—platelet-to-lymphocyte ratio; PNI—prognostic nutritional index; SII—systemic immune-inflammation index; SpO_2_—oxygen saturation; TMI—triponderal mass index.

**Table 2:** Spearman correlations of BMI, TMI, and AHI with clinical, polysomnographic, and laboratory parameters.

	BMI ρ (q)	TMI ρ (q)	AHI ρ (q)
Age (years) BMI (kg/m^2^) TMI (kg/m^3^)	0.357 (<0.001) N/A 0.960 (<0.001)	0.428 (<0.001) 0.960 (<0.001) N/A	0.223 (0.006) 0.515 (<0.001) 0.470 (<0.001)
ODI (events/h) Minimum SpO_2_ (%) Mean SpO_2_ (%) AHI (events/h)	0.403 (<0.001) −0.501 (<0.001) −0.545 (<0.001) 0.515 (<0.001)	0.354 (<0.001) −0.477 (<0.001) −0.532 (0.004) 0.470 (<0.001)	0.934 (<0.001) −0.770 (<0.001) −0.622 (<0.001) N/A
Haemoglobin (g/dL) Albumin (g/dL) CRP (mg/L) MLR MII PNI CAR CLR	−0.185 (0.018) −0.353 (<0.001) 0.506 (<0.001) −0.182 (0.021) 0.374 (<0.001) −0.385 (<0.001) 0.522 (<0.001) 0.445 (<0.001)	−0.275 (0.008) −0.373 (<0.001) 0.476 (<0.001) −0.177(0.026) 0.360 (<0.001) −0.381 (<0.001) 0.495 (<0.001) 0.423 (<0.001)	NS −0.483 (<0.001) 0.367 (<0.001) NS 0.319 (<0.001) −0.631 (<0.001) 0.397 (<0.001) 0.315 (<0.001)

Correlation coefficients are presented as Spearman’s rho (ρ). To control for multiple testing, *p*-values were adjusted using the Benjamini–Hochberg false discovery rate (FDR) method, and FDR-adjusted q-values are reported. NS indicates non-significant correlations after FDR correction (q ≥ 0.05). Ninety-five per cent confidence intervals are provided in Supplementary Table S1. AHI—apnea–hypopnea index; BMI—body mass index; CAR—C-reactive protein to albumin ratio; CLR—C-reactive protein to lymphocyte ratio; CRP—C-reactive protein; MII—multi-inflammatory index; MLR—monocyte-to-lymphocyte ratio; ODI—oxygen desaturation index; PNI—prognostic nutritional index; SpO_2_—oxygen saturation; TMI—triponderal mass index.

**Table 3:** Diagnostic performance of anthropometric indices and inflammation-related biomarkers for predicting severe obstructive sleep apnea.

Variable	AUC (95% CI)	Optimal Cut-Off	Sensitivity (%)	Specificity (%)	PPV (%)	NPV (%)	χ^2^ (*p*-Value)
Anthropometric indices							
BMI	0.834 (0.778–0.890)	≥33.3	70.5	83.1	58.9	89.1	61.2 (<0.001)
TMI	0.823 (0.764–0.882)	≥19.7	75.4	76.3	52.3	90.0	52 (<0.001)
Inflammation-related biomarkers							
CRP	0.771 (0.698–0.845)	≥3.8	72.1	77.4	52.4	89.0	48.7 (<0.001)
MII	0.744 (0.663–0.825)	≥2120	68.9	81.4	56.0	88.3	52.9 (<0.001)
CAR	0.791 (0.719–0.861)	≥0.77	77.0	75.7	52.2	90.5	53.7 (<0.001)
CLR	0.754 (0.675–0.834)	≥1.74	72.1	77.4	52.4	89.0	48.7 (<0.001)
PNI	0.994 (0.987–1.000)	≤48.1	95.1	97.7	93.5	98.3	202.9 (<0.001)
Albumin	0.836 (0.774–0.898)	≤4.49	83.6	76.8	55.4	93.2	69.9 (<0.001)

Receiver operating characteristic (ROC) curve analyses were performed to evaluate the diagnostic accuracy of each marker for identifying severe obstructive sleep apnea. The area under the curve (AUC) with 95% confidence intervals is reported for each parameter. Optimal cut-off values were determined using the Youden index. Sensitivity, specificity, positive predictive value (PPV), and negative predictive value (NPV) were calculated at the optimal cut-off. Associations between marker categories and severe OSA were assessed using chi-square (χ^2^) tests. BMI—body mass index; CAR—C-reactive protein to albumin ratio; CLR—C-reactive protein to lymphocyte ratio; CRP—C-reactive protein; MII—multi-inflammatory index; PNI—prognostic nutritional index; TMI—triponderal mass index.

**Table 4:** DeLong test-based comparisons of AUC values for anthropometric indices and inflammation-related biomarkers in predicting severe obstructive sleep apnea.

Comparison	AUC (Ref)	AUC (Comp)	ΔAUC	Z score	*p* Value
BMI vs. TMI	0.834	0.823	−0.011	1.20	0.229
BMI vs. CRP	0.834	0.771	0.063	1.48	0.138
BMI vs. CAR	0.834	0.790	0.044	1.06	0.289
BMI vs. CLR	0.834	0.754	0.080	1.77	0.078
BMI vs. PNI	0.834	0.994	−0.160	−5.55	<0.001

Pairwise comparisons of areas under the ROC curves were performed using DeLong’s test for correlated ROC curves derived from the same study population. Comparisons include anthropometric indices and individual inflammation-related biomarkers. ΔAUC represents the difference between the compared AUC values, and Z-scores indicate the standardized test statistic generated by DeLong’s method. A two-tailed *p*-value < 0.05 was considered statistically significant. AUC—area under the curve; BMI—body mass index; TMI—triponderal mass index; CRP—C-reactive protein; CAR—C-reactive protein–to–albumin ratio; CLR—C-reactive protein–to–lymphocyte ratio; PNI —prognostic nutritional index; ROC—receiver operating characteristic.

**Table 5:** Comparison of demographic, polysomnographic, and laboratory characteristics according to body mass index and triponderal mass index categories in patients with obstructive sleep apnea.

	BMI < 30 *n* = 70	BMI ≥ 30 *n* = 110	*p*-Value	TMI <17 *n* = 67	TMI ≥ 17 *n* = 113	*p*-Value
Male sex, *n* (%)	47 (67)	42 (38)	<0.001	52 (78)	37 (33)	<0.001
Age (years)	45.8 ± 10.5	51.6 ± 10.1	<0.001	43.2 ± 10	52.9 ± 8.9	<0.001
Total sleep time (min)	286 ± 61	280 ± 71	0.696	289 ± 61	278 ± 71	0.377
Sleep efficiency (%)	76 ± 16	76 ± 17	0.889	78 ± 14	79 ± 16	0.954
ODI (events/h)	16 (1–142)	26 (2–133)	0.002	16 (2–142)	24 (2–133)	0.003
Minimum SpO_2_ (%)	85 (60–92)	76 (50–91)	<0.001	84 (50–92)	77 (50–90)	<0.001
Mean SpO_2_ (%)	92 (77–96)	90 (58–96)	<0.001	92 (77–96)	90 (55–96)	<0.001
AHI (events/h)	13.6 (5.3–102.4)	27.5 (5.2–115.9)	<0.001	15.7 (5.4–97)	23.5 (5.2–110.7)	<0.001
Laboratory values, Leukocyte count * Neutrophil count * Lymphocyte count * Monocyte count * Haemoglobin (g/dL) Platelet count * MPV (fL) Albumin (g/dL)	7.3 (3.9–17.2) 4.2 (2.1–14.5) 2.3 (0.9–4.7) 0.6 (0.3–1.3) 15.4 ± 1.7 270 ± 70 10.1 (0.6–12.4) 4.68 ± 0.29	7.7 (4.4–13.2) 4.5 (2.0–9.7) 2.3 (0.6–6.2) 0.5 (0.2–1.4) 14.6 ± 1.8 255 ± 54 10.1 (0.5–12.5) 4.44 ± 0.41	0.529 0.729 0.499 0.722 0.007 0.309 0.208 <0.001	7.3 (3.9–17.2) 4.1 (2.1–14.5) 2.3 (0.9–5.3) 0.6 (0.3–1.3) 15.5 ± 1.8 265 ± 63 10 (0.6–12.4) 4.69 ± 0.31	7.9 (4.4–13.9) 4.5 (2.0–9.7) 2.3 (0.6–6.2) 0.5 (0.2–1.4) 14.6 ± 1.7 258 ± 60 10.2 (0.5–12.5) 4.47 ± 0.40	0.189 0.300 0.628 0.434 <0.001 0.593 0.872 <0.001
Biomarkers, CRP (mg/L) NLR PLR MLR SII MII PIV PNI CAR CLR	1.8 (0.2–9.7) 1.9 (0.8–8.7) 113 (55–283) 0.2 (0.1–0.7) 474 (210–2197) 950 (75–13,904) 290 (80–2175) 56.7 ± 6.8 0.39 (0.04–2.39) 0.8 (0.1–4.9)	4.2 (0.5–61.8) 1.8 (0.7–17.7) 107 (42–502) 0.2 (0.1–1.1) 465 (166–4873) 2038 (146–44,930) 239 (63–2652) 50.7 ± 9.3 1.50 (0.12–14.72) 2.0 (0.2–26.8)	<0.001 0.390 0.154 0.050 0.354 <0.001 0.388 <0.001 <0.001 <0.001	1.9 (0.2–7.1) 1.9 (0.9–8.7) 112 (55–283) 0.2 (0.1–0.7) 470 (210–2197) 944 (76–13,905) 279 (80–2175) 56.1 ± 7.3 0.4 (0.04–1.78) 0.8 (0.1–3.8)	4 (0.5–61.8) 1.9 (0.7–17.7) 108 (42–502) 0.2 (0.1–1.0) 466 (166–4873) 2030 (146–44,930) 240 (63–2652) 51.2 ± 9.4 0.88 (0.12–14.72) 2.0 (0.2–26.8)	<0.001 0.827 0.403 0.056 0.844 <0.001 0.557 <0.001 <0.001 <0.001

*: *x10^9/L.* Data are presented as mean ± standard deviation for normally distributed variables and as median (minimum–maximum) for non-normally distributed variables. Categorical variables are expressed as a number (percentage). Comparisons between BMI (<30 vs. ≥30 kg/m^2^) and TMI (<17 vs. ≥17 kg/m^3^) categories were performed using the independent-samples *t*-test or Mann–Whitney U test, as appropriate. Categorical variables were compared using the chi-square test. A two-tailed *p*-value < 0.05 was considered statistically significant. AHI—apnea–hypopnea index; BMI—body mass index; CAR—C-reactive protein to albumin ratio; CLR—C-reactive protein to lymphocyte ratio; CRP—C-reactive protein; MII—multi-inflammatory index; MLR—monocyte-to-lymphocyte ratio; MPV—mean platelet volume; NLR—neutrophil-to-lymphocyte ratio; ODI—oxygen desaturation index; PIV—pan-immune—inflammation value; PLR—platelet-to-lymphocyte ratio; PNI—prognostic nutritional index; SII—systemic immune-inflammation index; SpO_2_—oxygen saturation; TMI—triponderal mass index.

## Data Availability

The data supporting the findings of this study are available from the corresponding author upon reasonable request.

## Supplementary Materials

The following supporting information can be downloaded at: https://www.mdpi.com/article/10.3390/jcm15010273/s1, Table S1: Correlation analysis between BMI, TMI, and AHI with sleep and laboratory parameters in patients with OSA; Table S2: Spearman correlation coefficients with 95% confidence intervals between BMI, TMI, AHI, and clinical, polysomnographic, and laboratory parameters.

## Abbreviations

The following abbreviations are used in this manuscript:

OSA	Obstructive sleep apnea
AHI	The apnea–hypopnea index
BMI	Body mass index
OHS	Obesity hypoventilation syndrome
TMI	Triponderal mass index
CRP	C-reactive protein
SII	Systemic immune-inflammation index
NLR	neutrophil-to-lymphocyte ratio
PLR	Platelet-to-lymphocyte ratio
MLR	Monocyte-to-lymphocyte ratio
MII	Multi-inflammatory index
PIV	Pan-immune-inflammation value
PNI	Prognostic nutritional index
CLR	CRP-to-lymphocyte ratio
CAR	CRP-to-albumin ratio
PSG	Polysomnography
AASM	American Academy of Sleep Medicine
ROC	Receiver operating characteristic
ODI	Oxygen desaturation index
minSpO_2_	minimum oxygen saturation
meanSpO_2_	mean oxygen saturation
MPV	Mean platelet volume
PPV	Positive predictive value
NPV	Negative predictive value
AUC	Area under the curve

## References

[1] Lee W., Nagubadi S., Kryger M.H., Mokhlesi B. (2008). Epidemiology of Obstructive Sleep Apnea: A Population-based Perspective. Expert Rev. Respir. Med..

[2] Berry R.B., Budhiraja R., Gottlieb D.J. (2012). Rules for scoring respiratory events in sleep: Update of the 2007 AASM Manual for the Scoring of Sleep and Associated Events. Deliberations of the Sleep Apnea Definitions Task Force of the American Academy of Sleep Medicine. J. Clin. Sleep Med..

[3] Stelmach-Mardas M., Brajer-Luftmann B., Kuśnierczak M., Batura-Gabryel H., Piorunek T., Mardas M. (2021). Body Mass Index Reduction and Selected Cardiometabolic Risk Factors in Obstructive Sleep Apnea: Meta-Analysis. J. Clin. Med..

[4] Shahar E., Whitney C.W., Redline S., Lee E.T., Newman A.B., Nieto F.J., O’Connor G.T., Boland L.L., Schwartz J.E., Samet J.M. (2001). Sleep-disordered breathing and cardiovascular disease: Cross-sectional results of the Sleep Heart Health Study. Am. J. Respir. Crit. Care Med..

[5] Senaratna C.V., Perret J.L., Lodge C.J., Lowe A.J., Campbell B.E., Matheson M.C., Hamilton G.S., Dharmage S.C. (2017). Prevalence of obstructive sleep apnea in the general population: A systematic review. Sleep Med. Rev..

[6] Peterson C.M., Su H., Thomas D.M., Heo M., Golnabi A.H., Pietrobelli A., Heymsfield S.B. (2017). Tri-Ponderal Mass Index vs Body Mass Index in Estimating Body Fat During Adolescence. JAMA Pediatr..

[7] Wang X., Dong B., Ma J., Song Y., Zou Z., Arnold L. (2020). Role of tri-ponderal mass index in cardio-metabolic risk assessment in children and adolescents: Compared with body mass index. Int. J. Obes..

[8] Özyildirim C., Unsal E.N., Ayhan N.Y. (2023). Performance of triponderal mass index, body mass index z scores, and body mass index performance in the diagnosis of obesity in children and adolescents. Nutrition.

[9] Yetkin N.A., Baran B., Öztürk A., Oymak F.S., Gulmez İ., Tutar N. (2025). The Predictive Value of the Triponderal Mass Index in Adult Obstructive Sleep Apnea Severity. J. Clin. Pract. Res..

[10] Şahan T.D., Karakaya Z., Bora E.S., Efgan M.G., Topal F.E. (2025). Inflammatory indexes in emergency patients with hypertensive pulmonary Oedema: A critical insight. Am. J. Emerg. Med..

[11] Uzun M., Çalışkan-Yıldırım E., Gökcek S., Demir B., Karoğlu A. (2023). Prognostic Role of Pan Immune Inflammation Value and Systemic Inflammation Response Index in Small Cell Lung Cancer. Acta Oncol. Turc..

[12] Gupta L., Thomas J., Ravichandran R., Singh M., Nag A., Panjiyar B.K. (2023). Inflammation in Cardiovascular Disease: A Comprehensive Review of Biomarkers and Therapeutic Targets. Cureus.

[13] Bunul S.D., Alagoz A.N., Piri-Cinar B., Bunul F., Erdogan S., Efendi H.A. (2023). Preliminary Study on the Meaning of Inflammatory Indexes in MS: A Neda-Based Approach. J. Pers. Med..

[14] Güneş Z.Y., Günaydın F.M. (2024). The relationship between the systemic immune-inflammation index and obstructive sleep apnea. Sleep Breath..

[15] Nadeem R., Molnar J., Madbouly E.M., Nida M., Aggarwal S., Sajid H., Naseem J., Loomba R. (2013). Serum inflammatory markers in obstructive sleep apnea: A meta-analysis. J. Clin. Sleep Med..

[16] Baran B., Yetkin N.A., Tutar N., Türe Z., Oymak F.S., Gülmez İ. (2024). The Role of Sequentially Monitored Laboratory Values and Inflammatory Biomarkers in Assessing the Severity of COVID-19. Cureus.

[17] Öksüm Solak E., Baran Ketencioglu B., Cinar S.L., Kartal D., Borlu M. (2023). The role of new inflammatory markers in determining disease activation and severity in patients with hidradenitis suppurativa. Int. J. Dermatol..

[18] Hizli O., Cayir S., Coluk Y., Kayabasi S., Yildirim G. (2021). The novel indicators of moderate to severe sleep apnea: Fibrinogen to albumin ratio vs. CRP to albumin ratio. Eur. Arch. Otorhinolaryngol..

[19] Li J., Zhu N., Wang C., You L., Guo W., Yuan Z., Qi S., Zhao H., Yu J., Huang Y. (2023). Preoperative albumin-to-globulin ratio and prognostic nutritional index predict the prognosis of colorectal cancer: A retrospective study. Sci. Rep..

[20] Lundetræ R.S., Lehmann S., Saxvig I.W., Saeed S., Gislason T., Bjorvatn B. (2025). Severity of obstructive sleep apnea is related to C-reactive protein levels: The influence of comorbidities and self-reported sleep duration. Sleep Med..

[21] Sateia M.J. (2014). International classification of sleep disorders-third edition: Highlights and modifications. Chest.

[22] Esmaeili N., Gell L., Imler T., Hajipour M., Taranto-Montemurro L., Messineo L., Stone K.L., Sands S.A., Ayas N., Yee J. (2025). The relationship between obesity and obstructive sleep apnea in four community-based cohorts: An individual participant data meta-analysis of 12,860 adults. EClinicalMedicine.

[23] Du J., Yang S., Zeng Y., Ye C., Chang X., Wu S. (2024). Visualization obesity risk prediction system based on machine learning. Sci. Rep..

[24] Ued F., Castro M.J.S., Bardi L.R., Del Ciampo L., Martinez E.Z., Ferraz I.S., Contini A.A., Mello E., Nogueira-de-Almeida C.A. (2025). Triponderal Mass Index rather than Body Mass Index in discriminating high adiposity in Brazilian children and adolescents. Nutr. Hosp..

[25] Gul Siraz U., Hatipoglu N., Mazicioglu M.M., Ozturk A., Cicek B., Kurtoglu S. (2023). Triponderal mass index is as strong as body mass index in the determination of obesity and adiposity. Nutrition.

[26] Wali S.O., Abaalkhail B., AlQassas I., Alhejaili F., Spence D.W., Pandi-Perumal S.R. (2020). The correlation between oxygen saturation indices and the standard obstructive sleep apnea severity. Ann Thorac. Med..

[27] Uzair A., Waseem M., Bin Shahid A., Bhatti N.I., Arshad M., Ishaq A., Sajawal M., Toor Z., Ahmad O. (2024). Correlation Between Body Mass Index and Apnea-Hypopnea Index or Nadir Oxygen Saturation Levels in Patients with Obstructive Sleep Apnea. Cureus.

[28] Imani M.M., Sadeghi M., Farokhzadeh F., Khazaie H., Brand S., Dürsteler K.M., Brüh A., Sadeghi-Bahmani D. (2021). Evaluation of Blood Levels of C-Reactive Protein Marker in Obstructive Sleep Apnea: A Systematic Review, Meta-Analysis and Meta-Regression. Life.

[29] Koseoglu S., Ozcan K.M., Ikinciogullari A., Cetin M.A., Yildirim E., Dere H. (2015). Relationship Between Neutrophil to Lymphocyte Ratio, Platelet to Lymphocyte Ratio and Obstructive Sleep Apnea Syndrome. Adv. Clin. Exp. Med..

[30] Pau M.C., Zinellu A., Mangoni A.A., Paliogiannis P., Lacana M.R., Fois S.S., Mellino S., Fois A.G., Carru C., Zinellu E. (2023). Evaluation of Inflammation and Oxidative Stress Markers in Patients with Obstructive Sleep Apnea (OSA). J. Clin. Med..

[31] Faure P., Tamisier R., Baguet J.P., Favier A., Halimi S., Lévy P., Pépin J.L. (2008). Impairment of serum albumin antioxidant properties in obstructive sleep apnoea syndrome. Eur. Respir. J..

[32] Midik M.M., Gunenc D., Acar P.F., Karaca B.S. (2024). Prognostic Value of Blood-Based Inflammatory Markers in Cancer Patients Receiving Immune Checkpoint Inhibitors. Cancers.

[33] Zeng J., He J., Chen M., Li J. (2024). Association between mean platelet volume and obstructive sleep apnea-hypopnea syndrome: A systemic review and meta-analysis. PLoS ONE.

[34] Yan X., Wang L., Liang C., Zhang H., Zhao Y., Zhang H., Yu H., Di J. (2022). Development and assessment of a risk prediction model for moderate-to-severe obstructive sleep apnea. Front. Neurosci..

[35] Kanbay A., Tutar N., Kaya E., Buyukoglan H., Ozdogan N., Oymak F.S., Gulmez I., Demir R. (2013). Mean platelet volume in patients with obstructive sleep apnea syndrome and its relationship with cardiovascular diseases. Blood Coagul. Fibrinolysis.

