# Supplementary material for: Integrated Assessment of Obesity Indices and Novel Inflammatory Biomarkers in Predicting the Severity of Obstructive Sleep Apnea
Source: J Clin Med. 2025 Dec 29;15(1):273. doi: 10.3390/jcm15010273 (PMC12786763; doi:10.3390/jcm15010273)
Supplement: Supplementary file 1 [file jcm-15-00273-s001.zip › jcm-4040302-supplementary.pdf]

SUPPLEMENTARY

Supplementary Table S1. Correlation analysis between BMI, TMI, and AHI with sleep and laboratory parameters in patients with OSA

	BMI ρ (P)	TMI ρ (P)	AHI ρ (P)
Age (years)	0.357 (<0.001)	0.428 (<0.001)	0.223 (0.003)
ODI	0.403 (<0.001)	0.354 (<0.001)	0.934 (<0.001)
Minimum SpO_2 (%)	-0.501 (<0.001)	-0.477 (<0.001)	-0.770 (<0.001)
Mean SpO_2 (%)	-0.545 (<0.001)	-0.532 (0.002)	-0.622 (<0.001)
AHI (events/h)	0.515 (<0.001)	0.470 (<0.001)	N/A
BMI (kg/m^2)	N/A	0.960 (<0.001)	0.515 (<0.001)
TMI (kg/m^3)	0.960 (<0.001)	N/A	0.470 (<0.001)
Leukocyte count	0.095 (0.204)	0.092 (0.219)	0.189 (0.011)
Neutrophil count	0.058 (0.435)	0.068 (0.364)	0.147 (0.049)
Lymphocyte count	0.025 (0.736)	0.020 (0.786)	0.031 (0.678)
Monocyte count	-0.076 (0.312)	-0.083 (0.268)	0.093 (0212)
Haemoglobin	-0.185 (0.013)	-0.275 (0.005)	-0.048 (0.521)
Platelet count	-0.114 (0.126)	-0.097 (0.195)	-0.026 (0.726)
MPV	-0.089 (0.234)	-0.057 (0.449)	-0.228 (0.002)
Albumin	-0.353 (<0.001)	-0.373 (<0.001)	-0.483 (<0.001)
CRP	0.506 (<0.001)	0.476 (<0.001)	0.367 (<0.001)
NLR	-0.014 (0.850)	0.005 (0.946)	0.048 (0.523)
PLR	-0.140 (0.061)	-0.123 (0.099)	-0.056 (0.456)
MLR	-0.182 (0.015)	-0.177(0.017)	-0.048 (0.524)
SII	-0.081 (0.277)	-0.064 (0.393)	0.046 (0.541)
MII	0.374 (<0.001)	0.360 (<0.001)	0.319 (<0.001)
PIV	-0.097 (0.196)	-0.089 (0.237)	0.063 (0.400)
PNI	-0.385 (<0.001)	-0.381 (<0.001)	-0.631 (<0.001)

CAR	0.522 (<0.001)	0.495 (<0.001)	0.397 (<0.001)
CLR	0.445 (<0.001)	0.423 (<0.001)	0.315 (<0.001)

Correlation coefficients (Spearman's rho, ρ) are presented for the relationships between BMI, TMI, and AHI and selected hematologic and inflammatory markers. $p < 0.05$ was considered statistically significant. AHI — apnea–hypopnea index; BMI — body mass index; CAR — C-reactive protein to albumin ratio; CLR — C-reactive protein to lymphocyte ratio; CRP — C-reactive protein; MII — multi-inflammatory index; MLR — monocyte-to-lymphocyte ratio; MPV — mean platelet volume; NLR — neutrophil-to-lymphocyte ratio; ODI — oxygen desaturation index; OSA — obstructive sleep apnea; PIV — pan-immune—inflammation value; PLR — platelet-to-lymphocyte ratio; PNI — prognostic nutritional index; SII — systemic immune—inflammation index; SpO_2 — oxygen saturation; TMI — triponderal mass index.

Supplementary Table S2. Spearman correlation coefficients with 95% confidence intervals between BMI, TMI, AHI, and clinical, polysomnographic, and laboratory parameters

**Variable pair**	**Spearman's ρ**	**95% Confidence Interval**	**q-value (FDR)**
**BMI – Age**	0.357	0.24 to 0.46	<0.001
**TMI – Age**	0.428	0.31 to 0.53	<0.001
**AHI – Age**	0.223	0.09 to 0.35	0.006
**BMI – ODI**	0.403	0.29 to 0.51	<0.001
**TMI – ODI**	0.354	0.23 to 0.47	<0.001
**AHI – ODI**	0.934	0.91 to 0.95	<0.001
**BMI – Minimum SpO_2**	−0.501	−0.61 to −0.38	<0.001
**TMI – Minimum SpO_2**	−0.477	−0.59 to −0.35	<0.001
**AHI – Minimum SpO_2**	−0.770	−0.84 to −0.69	<0.001
**BMI – Mean SpO_2**	−0.545	−0.65 to −0.43	<0.001
**TMI – Mean SpO_2**	−0.532	−0.64 to −0.42	0.004
**AHI – Mean SpO_2**	−0.622	−0.71 to −0.52	<0.001
**BMI – CRP**	0.506	0.39 to 0.61	<0.001
**TMI – CRP**	0.476	0.35 to 0.58	<0.001

**AHI – CRP**	0.367	0.25 to 0.48	<0.001
**BMI – Albumin**	−0.353	−0.47 to −0.22	<0.001
**TMI – Albumin**	−0.373	−0.49 to −0.25	<0.001
**AHI – Albumin**	−0.483	−0.60 to −0.35	<0.001
**BMI – MII**	0.374	0.26 to 0.48	<0.001
**TMI – MII**	0.360	0.24 to 0.47	<0.001
**AHI – MII**	0.319	0.20 to 0.43	<0.001
**BMI – PNI**	−0.385	−0.50 to −0.26	<0.001
**TMI – PNI**	−0.381	−0.50 to −0.25	<0.001
**AHI – PNI**	−0.631	−0.71 to −0.53	<0.001
**BMI – CAR**	0.522	0.41 to 0.63	<0.001
**TMI – CAR**	0.495	0.38 to 0.60	<0.001
**AHI – CAR**	0.397	0.27 to 0.51	<0.001
**BMI – CLR**	0.445	0.33 to 0.55	<0.001
**TMI – CLR**	0.423	0.31 to 0.54	<0.001
**AHI – CLR**	0.315	0.19 to 0.43	<0.001

Correlation coefficients are presented as Spearman's rho (ρ) with corresponding 95% confidence intervals. To control for multiple testing, p-values were adjusted using the Benjamini–Hochberg false discovery rate (FDR) procedure, and FDR-adjusted q-values are reported. Only correlations remaining statistically significant after FDR correction are shown.

AHI — apnea–hypopnea index; BMI — body mass index; CAR — C-reactive protein to albumin ratio; CLR — C-reactive protein to lymphocyte ratio; CRP — C-reactive protein; ODI — oxygen desaturation index; OSA — obstructive sleep apnea; PNI — prognostic nutritional index; SII — systemic immune—inflammation index; SpO_2 — oxygen saturation; TMI — triponderal mass index.
